# Induction of the interleukin 6/ signal transducer and activator of transcription pathway in the lungs of mice sub-chronically exposed to mainstream tobacco smoke

**DOI:** 10.1186/1755-8794-2-56

**Published:** 2009-08-21

**Authors:** Sabina Halappanavar, Marsha Russell, Martin R Stampfli, Andrew Williams, Carole L Yauk

**Affiliations:** 1 Environmental Health Science and Research Bureau, Health Canada, Ottawa, Ontario, K1A 0L2, Canada; 2 Department of Pathology and Molecular Medicine, McMaster University, Hamilton, Ontario, L8N 3Z5, Canada; 3 BioStatistics and Epidemiology Division, Health Canada, Ottawa, Ontario, K1A 0L2, Canada

## Abstract

**Background:**

Tobacco smoking is associated with lung cancer and other respiratory diseases. However, little is known about the global molecular changes that precede the appearance of clinically detectable symptoms. In this study, the effects of mainstream tobacco smoke (MTS) on global transcription in the mouse lung were investigated.

**Methods:**

Male C57B1/CBA mice were exposed to MTS from two cigarettes daily, 5 days/week for 6 or 12 weeks. Mice were sacrificed immediately, or 6 weeks following the last cigarette. High density DNA microarrays were used to characterize global gene expression changes in whole lung. Microarray results were validated by Quantitative real-time RT-PCR. Further analysis of protein synthesis and function was carried out for a select set of genes by ELISA and Western blotting.

**Results:**

Globally, seventy nine genes were significantly differentially expressed following the exposure to MTS. These genes were associated with a number of biological processes including xenobiotic metabolism, redox balance, oxidative stress and inflammation. There was no differential gene expression in mice exposed to smoke and sampled 6 weeks following the last cigarette. Moreover, cluster analysis demonstrated that these samples clustered alongside their respective controls. We observed simultaneous up-regulation of *interleukin 6 *(*IL-6*) and its antagonist, *suppressor of cytokine signalling *(*SOCS3*) mRNA following 12 weeks of MTS exposure. Analysis by ELISA and Western blotting revealed a concomitant increase in total IL-6 antigen levels and its downstream targets, including phosphorylated signal transducer and activator of transcription 3 (Stat3), basal cell-lymphoma extra large (BCL-XL) and myeloid cell leukemia 1 (MCL-1) protein, in total lung tissue extracts. However, in contrast to gene expression, a subtle decrease in total SOCS3 protein was observed after 12 weeks of MTS exposure.

**Conclusion:**

Global transcriptional analysis identified a set of genes responding to MTS exposure in mouse lung. These genes returned to basal levels following smoking cessation, providing evidence to support the benefits of smoking cessation. Detailed analyses were undertaken for IL-6 and its associated pathways. Our results provide further insight into the role of these pathways in lung injury and inflammation induced by MTS.

## Background

Tobacco smoking is responsible for 90% of all lung cancers [1,2] and remains the second largest preventable cause of mortality and morbidity worldwide [3]. In addition to lung cancers, tobacco smoke is also linked to other respiratory diseases including chronic obstructive pulmonary disease (COPD) [4,5] and emphysema [6,7]. Despite the overwhelming evidence linking tobacco smoke to various respiratory pathologies, the percentage of smokers who develop any disease is relatively low [8].

The interaction between tobacco smoke and the pulmonary system involves complex molecular pathways. Using cells in culture, and animal and human models, it has been shown that various biological pathways (e.g., oxidative stress response, antioxidant activity, DNA repair, pro- and anti-inflammation) are generally induced in response to tobacco smoke. For example, increased levels of several oxidative stress markers in lung tissue have been reported in response to tobacco smoke including: 8-OHdG, 4-HNE [9], inducible nitric oxide synthase mRNA and endothelial nitric oxide synthase mRNA [10]. Exposure to cigarette smoke also causes changes in the expression of *heme oxygenase-1 *(*Hmox-1*), *c-myc, c-jun *and *c-fos *[11,12], induction of phase-I xenobiotic metabolism genes [13], increased expression and/or function of several proteinases including matrix metalloproteinases (MMP-1, -2, -9 and -14) [14-16] and enhanced NF-kB and AP-1 activity [17]. NF-kB and AP-1 regulate many of the inflammatory genes that are over-expressed in response to tobacco smoke [18,19]. These studies have considerably increased our understanding of the effects of smoking on health. However, these studies do not provide information on global changes in gene expression in target tissues. Tobacco smoke is a complex mixture of thousands of chemicals and exposure to it results in a highly complex molecular response. Consequently, the exact mechanisms by which smoking leads to disease in an individual, or the alterations in expression of specific genes that determine this susceptibility, are not entirely elucidated.

DNA microarray technology permits the simultaneous monitoring of thousands of transcripts expressed in a given cell or tissue type in a single experiment, and can be used to gain insight into complex molecular responses. Global transcriptional profiling has the potential to predict disease development and associated prognosis [20]. Several recent studies have used DNA microarray technology to delineate the molecular gene expression profiles that distinguish various subtypes and stages of lung cancer (reviewed in [21]). Others have documented gene expression profiles in various disease states including emphysema, COPD and cancers [22-25]. Many others have used cells in culture and tissues from animals exposed acutely or chronically to cigarette smoke to study the molecular pathways that may be involved in disease. In general, these studies report basic similarities in biological responses to tobacco smoke including the upregulation of antioxidants, and phase-I and phase-II xenobiotic metabolism genes. However, results generated from these studies reveal a large list of non-overlapping differentially expressed genes; these discrepancies necessitate additional studies to resolve differences and precisely define the mechanisms by which cigarette smoke exposure impacts gene expression profiles *in vitro *and *in vivo*, and to determine whether these changes reflect what is observed in human disease.

In this study, we used high-density DNA microarrays to examine global transcriptional changes in lung tissues derived from mice exposed to mainstream tobacco smoke (MTS) for 6 or 12 weeks, and following a period of smoking cessation. We identified genes that have been reported in other studies including *cytochrome P450, family 1 (Cyp1a1), Heme oxygenase (decycling)1 (Hmox1) *and *NAD(P)H dehydrogenase, quinine 1 (Nqo1)*. In addition, we observed induction of cytokine *interleukin 6 (IL-6) *mRNA and its antagonist, *suppressor of cytokine signalling (SOCS3)*, following 12 weeks of exposure to MTS. We also demonstrate an increase in total protein levels of IL-6 and its downstream targets basal cell-lymphoma extra large (BCL-XL) and myeloid cell leukemia 1 (MCL-1).

## Methods

### Animal care and husbandry

Exposures were conducted as described previously [26,27]. In brief, twenty mature (8–10 week old) male C57BL/6 × CBA F1 hybrid mice (The Jackson Laboratory. Bar Harbor, Maine) were exposed to MTS using a nose-only smoke exposure system [28] adapted for mice [29]. Mice were housed in a 12-h light-dark cycle with food and water *ad libitum*. Cages, food and bed were autoclaved. Mice were placed in individual exposure chambers (9 × 3 × 3 cm^3^) and were exposed to two cigarettes daily (1R3 reference cigarettes; Tobacco and Health Research Institute, University of Kentucky) at a rate of 0.08 litres per minute, 1 puff (20 ml) per 52 seconds, 5 days/week for a total of 6 or 12 weeks, including the 2 week lead-up period [27]. Control mice were placed in restrainers only. Animals were anesthetised with isoflurane and euthanised by exsanguation (3 hours after the last cigarette in the exposed group). Animal procedures were carried out under the guidelines of the Canadian Council on Animal Care and Procedures approved by the McMaster University Animal Research Ethics Board.

The selected dose of 2 cigarettes daily for 8 weeks has been shown to increase average serum cotinine levels to 150 ng/ml, which is consistent with the levels observed in regular active smokers (more than 100 ng/ml) [30].

### Bronchoalveolar Lavage

Mice were treated and sacrificed as described above. Bronchoalveolar lavage (BAL) was performed as previously described [31]. In brief, the lungs were dissected and the trachea was cannulated with a polyethylene tube (Becton Dickinson, Sparks, MD). The right lungs were lavaged twice with PBS (0.25 ml followed by 0.2 ml).

Approximately 0.3 ml of the instilled fluid was consistently recovered. Total cell counts were determined using a hemocytometer. After centrifugation, cell pellets were resuspended in PBS and smears were prepared by cytocentrifugation (Shandon Inc., Pittsburgh, PA) at 300 rpm for 2 min. Diff-Quik (Baxter, McGraw Park, IL) was used to stain all smears. Differential counts of BAL cells were determined from at least 500 leukocytes using standard hemocytological procedures to classify the cells as neutrophils, eosinophils, lymphocytes, or macrophages/monocytes.

### Tissue processing

The right lobe of the lung was lavaged for bronchoalveolar lavage fluid (BALF) and the left lobe of the lung was snap frozen in liquid nitrogen and stored at -80°C. For molecular analysis, the frozen left lung lobe was sliced randomly into two (upper and lower) halves. The upper half was used for RNA extraction. The lower half was further processed for total protein extracts.

### RNA extraction and purification

Total RNA was isolated using TRIzol reagent (Invitrogen) and purified using RNeasy Mini Kit (Qiagen). All RNA samples showed A260/280 ratios between 2.0 and 2.1. RNA integrity was determined using an Agilent 2100 Bioanalyzer (Agilent Technologies) and only high quality RNA (28S/18S > 1.8) was used for further analysis.

### Microarray hybridization

Individual total RNA samples (2.5 μg) from 40 mice (5 mice for each group, 4 treatment groups and 4 control groups) and universal reference total RNA (Stratagene) were used to synthesize double-stranded cDNA and cyanine labelled cRNA (experimental samples with Cyanine 5-CTP, and reference RNA with Cyanine 3-CTP; Perkin-Elmer Life Sciences) according to the manufacturer's instructions (Agilent Linear Amplification Kits, Agilent Technologies). Cyanine-labelled cRNA targets were in vitro transcribed using T7 RNA polymerase and purified by RNeasy Mini Kit (Qiagen). Five micrograms of each labelled cRNA was hybridized to Agilent 4121A oligonucleotide microarrays (Agilent Technologies) at 60°C overnight. Arrays were washed and scanned on a ScanArray Express (Perkin-Elmer Life Sciences), and data were acquired with ImaGene 5.5 (BioDiscovery).

### Statistical analysis of microarray data

A blocked factorial design [32] was used to analyse lung microarray data. The factors in the data included treatment (control, exposed), duration of exposure (6 weeks, 12 weeks) and a break period (0 weeks, 6 weeks). The design was blocked using the date of hybridization and the date of exposure [33]. Five biological replicates per condition were used for a total of 40 microarrays.

The background for each array was measured using the (-)3xSLv1 probe. Spots with median signal intensities less than the trimmed mean (trim = 5%) plus three trimmed standard deviations of the (-)3xSLv1 probe were flagged. The total number of flagged spots, the median signal intensity and standard deviation for the (-)3xSLv1 probe for each array were recorded. Other array level summary statistics included the median signal to noise ratio (log_2 _scale) for each channel. This information was used to help identify microarrays with poor data quality.

The data were normalized using a MAANOVA library [34] in R [35]. Ratio intensity plots and heat maps for the raw and normalized data were constructed using R [35]. Through inspection of the dendrogram one outlier was identified. This microarray also had high background and thus this sample was then removed from all subsequent analyses.

Differentially expressed genes between the control and treated groups within time points were determined using the MAANOVA library [34] in R. The main effects in the model included treatment, duration of exposure and break period, as well all two-way and the three-way interaction. This model was applied to the log_2 _of the relative intensities. The Fs statistic [36], a shrinkage estimator for the gene-specific variance components, was used to test main effects, interactions and pair-wise comparisons. The p-values for all statistical tests were estimated by the permutation method using residual shuffling, followed by adjustment for multiple comparisons by using the false discovery rate (FDR) approach [37].

The group means for the fold change calculation were based on the least-square means. Least-square means are a function of the ANOVA model parameters and are adjusted for the other factors in the model such as date of hybridization.

### Validation of microarray results by real-time polymerase chain reaction (RT-PCR)

Primers were designed using Beacon design 2.0 (Premier BioSoft International) and are available upon request. Approximately 2.5 μg of total RNA per sample was reverse transcribed and RT-PCR was performed in duplicate using an iCycler IQ real-time detection system (Bio-Rad) as described in [38]. Threshold cycle values were averaged. Gene expression levels were normalized to the ubiquitin gene, which was stable on the DNA microarray. PCR efficiency was examined using the standard curve for each gene. Primer specificity was assured by the melting curve for each gene. A student's t-test was used for statistical evaluation.

### Preparation of tissue protein extracts and Western blotting

The lower half of the frozen left lobe was homogenized in lysis buffer (5 M HEPES, pH 7.5, 5 M NaCl, 10% Glycerol, 1% Triton X-100, 2 M EGTA, 1 M MgCl_2_, 0.5 M NaF, 0.2 M sodium pyrophosphate, protease inhibitor cocktail tablets (Roche Applied Science) and centrifuged. The supernatant was quantified for protein content using a Bradford protein assay reagent kit (Bio-Rad). Approximately 200 μg of total protein was extracted from each individual mouse (n = 5/treatment group) from one treatment group, and subsequently pooled to make one sample. The protein content of each pooled sample was quantified again using a Bradford protein assay kit.

For Western blotting, 30 μg of individual (data not shown) or pooled total protein was immunoblotted on 8–12% SDS-PAGE gels and analysed using antibodies against SOCS3, BCL-XL, Stat3, Stat3 phospho, MCL-1, Gp-130 and JAK-1 (Santa Cruz Biotechnologies). Signals were detected using ECL Plus reagent (GE Health Sciences). Membranes were erased and reprobed with anti-actin antibody for normalizing purposes. Band intensities were determined by averaging the densitometric readings from three independent experiments. Protein levels were normalized to the actin levels present in each sample.

### Total IL-6 Immunoassay

The Quantikine Mouse IL-6 immunoassay (R&D systems) was used to measure total IL-6 in lung tissue homogenates. The assay was conducted according to the manufacturer's instructions. In brief, 50 μl of assay diluent and known quantities of samples (15–30 μg total tissue homogenates) and controls (0–500 pg/ml mouse IL-6 standard, supplied by the company) were loaded onto a microplate pre-coated with mouse IL-6 specific antibody. The plate was incubated at room temperature for 2 hours, then unbound IL-6 was removed by washing five times with wash buffer. An enzyme-linked polyclonal antibody specific to mouse IL-6 was then added to each well and incubated for 2 hours at room temperature. Plates were washed as described above to remove any unbound enzyme conjugate. One-hundred microliters of substrate solution was added to each well and incubated in the dark for 30 minutes at room temperature. The reaction was quenched by adding 100 μl of stop solution to individual wells. Optical Density for each well was determined at 450 nm using a microtiter plate spectrophotometer with the correction wavelength set at 540 or 570 nm.

## Results

### General overview of expression profiles and validation of microarray results

Complete DNA microarray data are available at NCBI , GSE12930. Approximately 70% of the 22,000 transcripts on the array were expressed (where expressed is defined as at least 4 out of 5 samples with signal intensities above background in at least one experimental condition). Differentially expressed genes were identified using MAANOVA; values were considered significantly different from control values when FDR adjusted p-values were less than 0.05.

Statistically significant differential gene expression was identified for 79 genes between smoke-exposed groups and matched controls at either 6 or 12 week time points (Table-1; 52 up-regulated and 27 down-regulated compared to sham controls). These genes belong to various biological processes including xenobiotic metabolism, oxidative stress, glutathione metabolism, inflammatory pathways and others. Genes that are implicated in xenobiotic metabolism, such as *Cyp1a1*, (24-fold), *cytochrome P450, family 1, subfamily b, polypeptide 1 (Cyp1b1*, 7-fold), *Nqo1*, (3-fold) and *aryl-hydrocarbon receptor repressor (Ahrr*, 3-fold), showed the greatest increase in expression in smoke-exposed groups (Table-1). Genes that showed decreased expression in MTS exposed groups include *nuclear antigen SP-100 *(*Sp100*),*T-cell lymphoma invasion and metastasis 1 *(*Tiam1*) and *solute carrier family 12, member 1 *(*Slc12a1*) (between 1.3–1.5-fold down-regulation for all), associated with transcription, signal transduction and transport functions respectively (Table-1). We also observed time-dependent increases in the expression of some genes including *IL-6 *and *SOCS3*, which were significantly induced only after 12 weeks of exposure to smoke. Table-1 summarizes the genes, fold induction and related functions. In addition, we noted that changes in gene expression were transient and returned to basal levels when MTS exposure was discontinued for all genes (Table-1, columns 2 and 4).

**Table 1:** Mouse Lung: Significantly differentially expressing genes

**Description**	*Fold change			
**Xenobiotic Metabolism**	**1**	**2**	**3**	**4**
Cytochrome P450, family 1(Cyp1a1)	24.20	-1.02	26.93	-1.11
Cytochrome P450, family 1(Cyp1b1)	7.26	1.05	7.79	1.05
Aryl-hydrocarbon receptor repressor (Ahrr)	2.46	-1.04	3.92	1.13
NAD(P)H dehydrogenase, quinone 1 (Nqo1)	2.64	1.06	3.70	-1.00
**Redox Balance**				
Carbonyl reductase 3 (Cbr3)	3.42	1.11	3.54	-1.03
homolog (S. cerevisiae) (Srxn1)	1.93	1.04	3.23	-1.06
Alcohol dehydrogenase 7 (class IV)(Adh7)	2.15	-1.02	3.22	1.08
Aldehyde dehydrogenase family 3, subfamily A1 (Aldh3a1)	3.08	1.09	3.08	1.07
Aldo-keto reductase family 1	2.03	1.03	2.34	1.09
Thioredoxin reductase 1 (Txnrd1)	2.06	1.03	2.32	1.05
Aldehyde oxidase 1 (Aox1)	1.24	1.08	2.23	1.16
**Glutathione metabolism**				
Glutamate-cysteine ligase, catalytic subunit (Gclc)	2.40	1.00	4.06	1.30
Glutamate-cysteine ligase, modifier subunit (Gclm)	1.87	1.00	2.14	-1.04
Glutathione reductase 1 (Gsr)	1.31	1.06	1.34	-1.00
Glutathione S-transferase, theta 2 (Gstt2)	-1.38	1.08	-1.05	1.12
**Oxidative stress, Inflammatory pathways**				
Heme oxygenase (decycling) 1 (Hmox1)	2.32	-1.09	4.41	-1.08
Interleukin 6 (Il6)	1.06	1.38	2.48	1.12
Prostaglandin-endoperoxide synthase 2 (Ptgs2)	1.87	1.47	2.91	1.13
Suppressor of cytokine signaling 3 (Socs3)	1.41	1.16	1.70	1.05
Lectin, galactose binding, soluble 3 (Lgals3)	1.55	-1.09	1.53	-1.11
Tumor necrosis factor, alpha-induced protein 2 (Tnfaip2)	1.53	1.16	1.30	-1.05
Paraoxonase 3 (Pon3)	1.31	1.01	1.43	1.14
**Molecular chaperones**				
Heat shock protein 1A (Hspa1a)	2.37	-1.22	2.69	-1.46
Heat shock 70 kDa protein 4 like (Hspa4l)	1.52	-1.01	1.79	-1.03
**Development**				
keratin complex 1, acidic, gene 19 (Krt1-19)	1.60	1.05	1.68	-1.02
ADP-ribosylation factor related protein 2 (Arfrp2)	-1.60	-1.02	-1.40	-1.08
Procollagen, type V, alpha 1 (Col5a1)	-1.24	1.07	-1.38	-1.01
**Signal transduction pathways**				
Mitogen-activated protein kinase kinase kinase 6 (Map3k6)	1.44	-1.12	1.79	-1.17
PH domain and leucine rich repeat protein phosphatase	1.64	-1.03	1.12	-1.04
Breast cancer anti-estrogen resistance 3 (Bcar3)	1.30	-1.10	1.56	-1.02
Inosine triphosphatase (nucleoside triphosphate pyrophosphatase) (Itpa)	1.22	1.08	1.06	1.01
Cytoplasmic tyrosine kinase, Dscr28C related (Drosophila) (Tec)	-1.25	-1.03	1.04	1.08
Serine/threonine kinase 4 (Stk4)	1.47	1.01	-1.04	-1.17
Immediate early response 3 (Ier3)	1.42	1.03	1.47	-1.23
Sphingosine kinase 1 (Sphk1)	1.23	-1.24	1.51	-1.26
Platelet derived growth factor receptor, beta polypeptide (Pdgfrb)	-1.42	1.02	-1.50	1.07
**Transport**				
Chloride channel 2 (Clcn2)	-1.39	1.05	-1.07	1.06
Solute carrier family 12, member 1 (Slc12a1)	-1.57	1.02	-1.04	1.26
**Transcription**				
Nuclear antigen Sp100 (Sp100)	-1.55	1.11	-1.32	1.00
Chromatin accessibility complex 1 (Chrac1)	1.47	1.07	1.03	-1.11
X-box binding protein 1 (Xbp1)	1.36	-1.14	1.18	-1.10
TAF9 RNA polymerase II, TATA box binding protein (TBP)-associated	1.29	1.07	1.30	1.04
Transcription factor E2a (Tcfe2a)	-1.29	-1.02	-1.35	-1.08
**Structure**				
LIM-domain containing, protein kinase (Limk1)	1.49	1.06	1.30	-1.14
DIX domain containing 1 (Dixdc1)	-1.31	1.20	-1.41	1.16
Ankyrin 3, epithelial (Ank3), transcript variant 1	-1.14	1.09	-1.27	1.10
**Oncogene, tumour suppressor**				
B-cell leukemia/lymphoma 3 (Bcl3)	1.73	1.00	2.13	-1.15
T-cell lymphoma invasion and metastasis 1 (Tiam1)	-1.19	1.13	-1.45	1.10
Kruppel-like factor 9 (Klf9)	1.02	-1.47	1.01	-1.32
**Others**				
Growth arrest and DNA-damage-inducible 45 gamma (Gadd45g)	1.21	-1.01	1.95	-1.18
Enoyl Coenzyme A hydratase domain containing 3 (Echdc3)	-1.50	1.13	-1.12	1.13
Cathepsin D (Ctsd)	1.54	-1.05	1.46	1.03
Angiomotin like 2 (Amotl2)	1.47	1.15	-1.02	-1.02
Glucose-6-phosphate dehydrogenase X-linked (G6pdx)	1.42	-1.00	1.20	-1.05
TG interacting factor (Tgif)	-1.06	1.07	1.33	-1.01
Nucleolar protein 5A (Nol5a)	1.31	1.02	1.03	-1.10
Protein-O-mannosyltransferase 1 (Pomt1)	-1.24	1.02	-1.03	1.05
Chitinase 3-like 3 (Chi3l3)	3.19	-1.12	2.91	1.25
Leukotriene C4 synthase (Ltc4s)	1.81	-1.06	1.19	1.10
ATPase, H+ transporting, V0 subunit D, isoform 2 (Atp6v0d2)	1.76	-1.15	1.58	-1.13
Cold inducible RNA binding protein (Cirbp)	-1.48	1.14	-1.58	1.06
**Unknown and RIKENS**				
Lung-inducible neuralized-related C3HC4 RING domain protein (Lincr)	1.45	1.08	1.22	-1.04
Selenocysteine lyase (Scly)	-1.29	1.14	-1.26	1.00
RIKEN cDNA B230118H07 gene	-1.07	1.08	-1.29	1.12
RIKEN cDNA 1110019L22 gene	1.13	1.02	-1.22	-1.27
Zinc finger CCCH type, antiviral 1 (Zc3hav1)	-1.33	-1.06	-1.18	-1.02
RIKEN cDNA 9830165K03	-1.40	1.12	-1.18	1.02
RIKEN cDNA 2310007H09 gene	-1.38	1.11	-1.07	1.11
mRNA for mKIAA1201 protein	-1.31	1.09	-1.36	1.00
Oolfactory receptor 1286 (Olfr1286)	-1.87	1.17	1.06	-1.04
Procollagen, type III, alpha 1 (Col3a1)	-1.59	-1.05	-1.77	1.17
RIKEN cDNA 1190002H23 gene	-1.25	-1.14	1.73	-1.06
4 days neonate male adipose cDNA, RIKEN	1.23	-1.54	1.15	-1.70
Roundabout homolog 2 (Drosophila) (Robo2)	-1.66	1.04	-1.18	1.22
Adult male urinary bladder cDNA, RIKEN	1.11	1.12	-1.04	^-1.64^
RIKEN cDNA 1700012B18 gene	1.33	-1.23	2.25	-1.21
Btg3 associated nuclear protein (Banp)	-1.36	-1.48	1.16	1.06
RIKEN cDNA 9130213B05 gene	1.39	1.00	1.52	-1.01
E74-like factor 3 (Elf3)	1.51	1.03	1.47	^1.06^

* **1**. 6 weeks smoke, **2**. 6 weeks smoke followed by 6 weeks break, **3**. 12 weeks smoke

**4**. 12 weeks smoke followed by 6 weeks break

**Figure 1:**
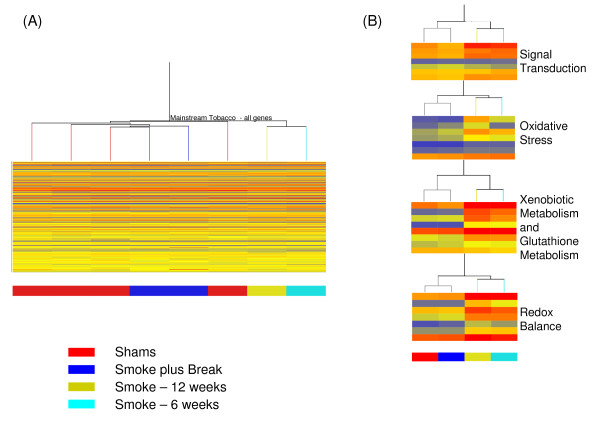
**Hierarchical cluster analysis of samples grouped into the 4 control and 4 treatment groups**. A. Cluster analysis of all genes present on the chip. B. Heat maps represent select genes (statistically differentially expressing, Table-1) from signal transduction, redox balance, oxidative stress and xenobotic metabolism processes. Red bars represent high expression levels, blue represent low expression levels and yellow bars are similar to the normalized median gene expression values.

**Figure 2:**
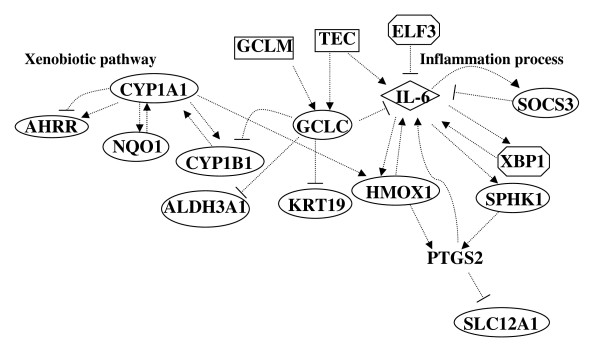
**Gene networks derived from direct interactions mined in PathWay Studio using the list of significantly differentially expressed genes in the treated groups**. The arrow heads indicate positive regulation. --| indicates negative regulation.

Hierarchical cluster analysis was conducted on the average expression for individual genes within each experimental condition. Clustering was based on the expression pattern of all genes on the array (22,000, Figure 1A) or using genes that are statistically significantly differentially expressing from select functional groups (Figure 1B). The main branch of the tree split the samples collected immediately following smoke exposure from the other groups (Figure 1A). Lungs samples from mice exposed to smoke and collected following a 6 week break period clustered alongside controls. Pathway Studio (version-5, Ariadne Genomics Inc.) was used to identify specific biological pathways associated with the differentially expressed genes. One single network was generated using the direct interaction algorithm for the differentially expressed genes in the MTS exposure group. This network included two core modules (Figure 2) relating to the xenobiotic response pathway, and inflammation, cell survival and proliferation pathways.

**Figure 3:**
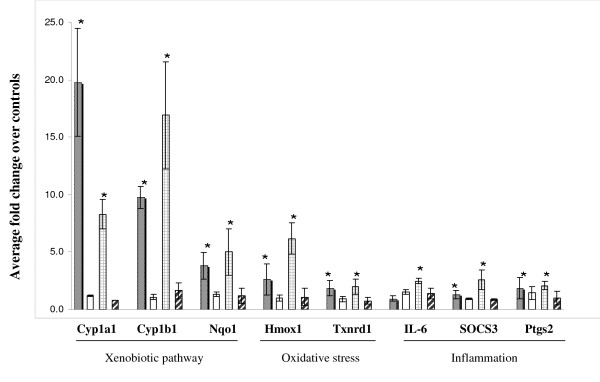
**Validation of microarray results. Data are presented as fold change relative to sham controls. (n = 5 mice/group, ± SEM)**. Gray bars: 6 weeks smoke. White bars: 6 weeks smoke + 6 weeks break. Bars with hatched lines: 12 weeks smoke. Bars with diagonal lines: 12 weeks smoke + 6 weeks break. * indicates significant results.

Real time RT-PCR was performed to validate the expression levels of select differentially expressed genes identified by microarray analyses on the same RNA samples used for microarray analysis (Figure 3). Results confirmed the up-regulation of *Cyp1A1, Cyp1B1, Nqo1, thioredoxin reductase 1 (Txnrd1), Hmox1, IL-6, SOCS3 *and *prostaglandin-endoperoxide synthase 2 (Ptgs2) *in 6 and 12 weeks MTS exposed samples. In concordance with the microarray results, changes were mostly reversed following smoking cessation. Gene expression changes for *Cyp *genes, *IL-6 *and *SOCS3 *were also confirmed by RT-PCR in lung tissues of the same strain of female mice exposed to MTS using an identical exposure system and experimental design (data not shown).

### BALF inflammatory profile

**Figure 4:**
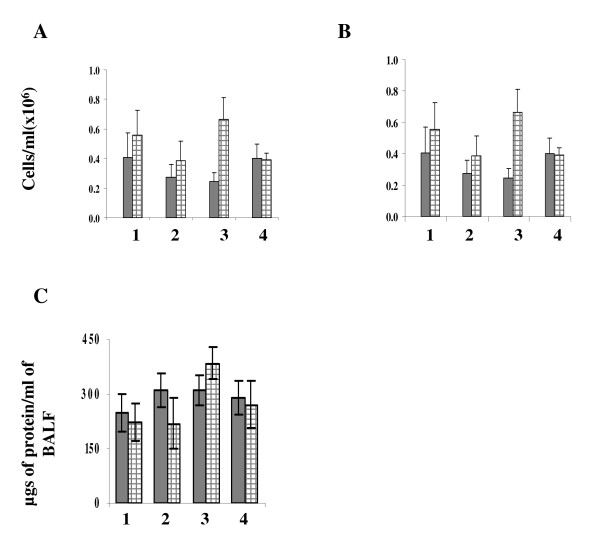
**Inflammatory profile in the bronchoalveolar lavage (BAL) fluid. Sham (gray bars) and MTS exposed (bars with hatched lines)**. BAL was performed at the indicated time points and total BAL cells (A), mononuclear cells (B) and total amount of protein (C) was analysed. Error bars represent SEM, n = 5/group. 1: 6 weeks smoke. 2: 6 weeks smoke + 6 weeks break. 3: 12 weeks smoke. 4: 12 weeks smoke + 6 weeks break.

To assess the inflammatory response to tobacco smoke exposure in mouse lungs, inflammatory cell counts were performed on the BALF. Total BALF cell count (Figure 4A) and total number of mononuclear cells (Figure 4B) each increased by approximately 1.5 fold at 6 weeks, and increased by 2 and 3 fold respectively following 12 weeks of cigarette smoke exposure. There was also a subtle increase in the total amount of protein at 12 weeks (Figure 4C). However, no changes were observed in total number of neutrophils (data not shown).

### Activation of IL-6/signal transducer and activator of transcription (Stat) pathway

**Figure 5:**
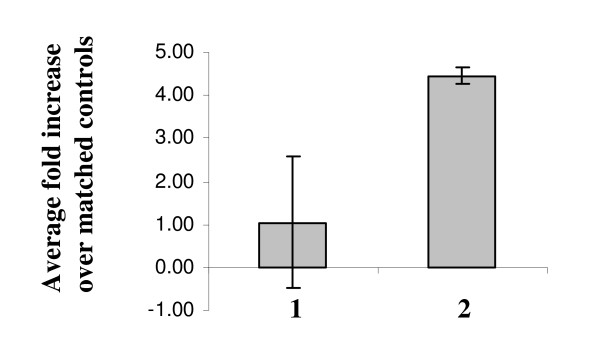
**Total immunoreactive IL-6 in lung tissue extracts**. Data represent fold change relative to sham controls (n = 5, ± SEM). 1: 6 weeks smoke. 2: 12 weeks smoke.

IL-6 has been implicated in the promotion of inflammation, cell proliferation and differentiation [39]. Since our microarray data showed induction of *IL-6 *mRNA in response to cigarette smoke, we examined IL-6 protein levels in lung tissue extracts and BALF by ELISA. In alignment with the microarray results (Table-1), there was no increase in total IL-6 antigen in samples collected following 6 weeks exposure to MTS (Figure 5). However, at 12 weeks, total IL-6 antigen levels increased 4-fold compared to matched controls. In contrast, IL-6 was below detection levels in BALF (data not shown, not enough repeats).

**Figure 6:**
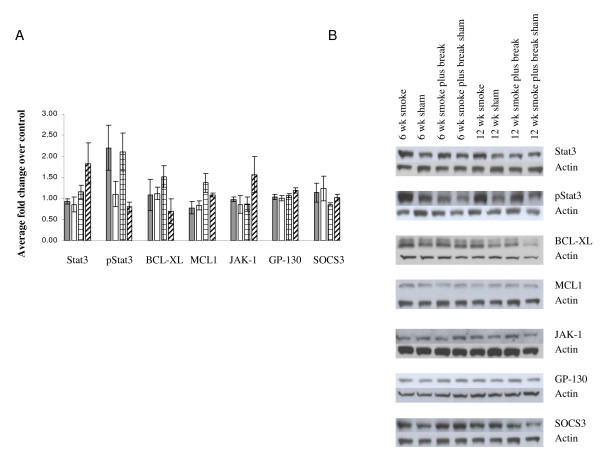
**(A) Quantification of Western blot for select proteins in lung tissue extracts**. Data are presented as fold change relative to sham controls (n = 5 mice/group, ± SEM). Gray bars: 6 weeks smoke. White bars: 6 weeks smoke + 6 weeks break. Bars with hatched lines: 12 weeks smoke. Bars with diagonal lines: 12 weeks smoke + 6 weeks break. (B) Gel photo of Western blots for each protein quantified.

Activation of the Janus family of tyrosine kinase (JAK)/Stat pathway is critical for IL-6 signalling. IL-6-induced activation of Stat3 is believed to contribute to cell proliferation and anti apoptotic activities by inducing BCL-XL and MCL-1 expression. To explore the potential repercussions of increased expression of IL-6 at 12 weeks following MTS exposure, we examined the levels of protein expression of some downstream targets of IL-6 by Western blotting. While there was no significant change in the total amount of Stat3 protein, an increase in phosphorylated Stat3 (2-fold) was observed as early as 6 weeks. This increase was sustained at 12 weeks. The increase in phosphorylated Stat3 was accompanied by a concomitant increase in total levels of BCL-XL and MCL-1 protein at 12 weeks only. We then investigated total levels of JAK-1 and Gp-130 proteins and their phosphorylation status in lung tissue extracts. However, we found no change in total levels of either protein (Figure 6). In addition, we did not see any differences in levels of phosphorylation of JAK-1 or Gp-130 among the treatment groups (data not shown).

Lastly, we analysed SOCS3 protein levels. There was no change in total SOCS3 antigen levels following 6 weeks exposure to MTS. However, levels of SOCS3 protein were reduced slightly following 12 weeks exposure relative to shams. Despite an increase in *SOCS3 *mRNA at 12 weeks, total SOCS3 protein levels decreased, suggesting the involvement of post transcriptional regulators of SOCS3. It has been reported that the inhibitory activity of SOCS3 on IL-6 is antagonized by its phosphorylation. However, we were unable to detect any phosphorylated SOCS3 (data not shown), suggesting that other inhibitory mechanisms could be involved.

## Discussion

Tobacco smoke is a complex mixture of gases, suspended particulate material and condensed organic compounds. Exposure to MTS results in a very complex physiological response involving numerous genes and signalling pathways. Global gene expression analysis using high density microarrays provides the unique advantage of studying multi-phasic responses of lungs to cigarette smoke. Changes in gene expression are presumed to occur in the early stages of disease development and may therefore be useful for predicting and understanding eventual disease outcome.

We carried out global transcriptional profiling of lung samples from adult male mice exposed sub-chronically to MTS and sacrificed 3 hours following the final cigarette exposure or subsequent to 6 weeks of smoking cessation. Our results revealed statistically significant changes in gene expression for 79 genes. Of these, 52 genes (69%) represented increased expression following either 6 or 12 weeks of MTS exposure (Table-1). Cluster analysis of all genes on the array, and of differentially expressed genes only, revealed that the primary factor influencing transcription profile was exposure to MTS. Inside the two main branches, all samples clustered tightly within their respective groups. Exposed lung samples collected following the cessation period clustered on the same main branch as the control groups, and alongside (but on different branches from) their respective controls (Figure 1A). No genes were statistically significantly differentially expressed in the groups of mice exposed to smoke followed by a 6 week break, compared with time-matched shams. Therefore, a break of 6 weeks generally led to a return of gene expression to basal levels, providing biological evidence to support the benefits of smoking cessation on pulmonary health. Similar results showing reversal of changes in gene expression following smoking cessation have been documented in rat lungs [40].

Several genes identified in this study (Table-1) have also been described by others. These include genes involved in the metabolism of polycyclic aromatic hydrocarbons present in the smoke, oxidative stress, redox regulation and inflammation [25,41-45], suggesting commonality in responses to tobacco smoke regardless of study design and species. However, the magnitude and timing of expression of select genes within these pathways differs between studies. Our results revealed well-orchestrated multi-phase responses in lungs to tobacco smoke. For example, we found: (a) early and sustained (Cyp genes, 6 and 12 weeks) up-regulation of genes involved in the xenobiotic metabolism pathway; (b) increases in the expression of genes that are implicated in oxidative stress (*Hmox-1*, *Txrnd1*) over time; and (c) up-regulation of genes implicated in inflammation processes (*IL-6*, *SOCS3*) at the later time point exclusively. Paradoxically, both cytokine *IL-6 *and its antagonist, *SOCS3*, were up-regulated simultaneously at 12 weeks (Table-1), which has not been reported by previous studies.

Elevated IL-6 concentrations have been associated with infection and inflammation in a variety of disease conditions [46-48]. IL-6 plays an important role in chronic diseases by inducing acute phase proteins, producing autoantibodies, and regulating local inflammatory events and associated systemic symptoms [49-51]. IL-6 is therefore targeted for therapeutic management of infectious and inflammatory diseases [52,53]. In a classical IL-6 signalling pathway, cytokine IL-6 secreted by monocytes or macrophages binds to its cell surface receptor IL-6Rα. A homodimer of signal transducer Gp-130 is then recruited to the IL-6-IL-6Rα complex and JAK-1 is activated. Once activated, JAK-1 in turn activates transcription factor Stat3 by phosphorylation. Phosphorylated Stat3 is dimerized and translocated to the nucleus where it induces transcription of a series of genes that include Bcl2, BCL-XL, Junb and MCL-1, all of which promote growth and inhibit apoptosis [54,55]. Therefore, our findings of transcriptional upregulation of IL-6 in mouse lung following 12 weeks of MTS exposure, along with concomitant increases in IL-6 antigen, phosphorylated Stat3, MCL-1 and BCL-XL protein levels, support the classic IL-6 signalling pathway. In this model, IL-6 plays a protective role via promotion of proliferation and inhibition of apoptosis. Our results are substantiated by other similar studies in the literature. For example, the IL-6/STAT3 pathway mediates survival of human bronchial epithelial cells following cigarette smoke condensate -induced DNA damage [56]. Over-expression of the activated form of STAT3 in alveolar type II epithelial cells leads to pulmonary inflammation and tumorigenesis in STAT3 transgenic mice [57]. Increased mRNA expression of STAT3 and a few of its downstream targets have previously been observed in lung tissues of smokers suffering from COPD [58].

In an alternate signalling pathway, called trans-signalling, free serum IL-6 can bind to soluble IL-6Rα and the resulting complex can signal any cell (many of which are unresponsive to IL-6 alone) that expresses Gp-130 at its surface. IL-6 trans-signalling is responsible for most of the harmful effects of IL-6 related to inflammation. In many chronic inflammatory diseases, including chronic inflammatory bowel disease, peritonitis, rheumatoid arthritis, asthma, as well as colon cancer, IL-6 trans-signalling promotes transition from acute to chronic inflammation and thereby aids in the maintenance of a disease state [59].

In many local acute inflammatory stages, early leukocyte recruitment is characterized by accumulation of neutrophils, marking the initiation of inflammation. At later stages of the inflammation process, these neutrophils are replaced by a more sustained population of mononuclear cells. It has been suggested that the switch in the leukocyte recruitment pattern determines clearance of inflammation and restoration of tissue homeostasis [60]. IL-6 regulates this transition during acute inflammatory processes and therefore plays a role in the resolution of acute inflammation [61,62]. However, in chronic conditions, altered IL-6 expression or function may lead to loss of this delicate balance and subsequent disease progression.

Tobacco smoke has been shown to have a suppressive effect on inflammatory mediators such as IL-6 in some models. In BALF and alveolar macrophages derived from rats exposed acutely to tobacco smoke, degradation of IL-6 and decrease in its activity was observed [63,64]. In contrast, IL-6 levels in human blood were unaffected by tobacco smoke exposure [65] suggesting that the acute effects of MTS on IL-6 is local or that other cells in blood may mediate the IL-6 levels.

Induction of IL-6 at 12 weeks in our model suggests that continued exposure of these mice to MTS may induce other pathways that initiate inflammatory processes. Alternatively, increased expression of molecules such as Txnrd1 and Hmox-1, both of which are up-regulated in response to MTS in our model (Table-1, Figure 3) may counteract inflammatory signals. In C57BL6/J mice, intra peritoneal injection of recombinant human Txnrd1 or in human Txnrd1 transgenic mice resulted in a reduction in cigarette smoke-induced lung inflammation and emphysema [43]. Hmox-1 is known to protect against inflammation. In a study where mice were exposed to cigarette smoke in presence of Hmox-1 inducers, Hmox-1 induction prevented B-cell infiltrates, similar to the lymphoid follicles found in COPD patients [66], and is thus suggested to play a role in COPD development. COPD is a disease driven by chronic inflammation. We have previously demonstrated down-regulation of plasminogen activator inhibitor-1 (PAI-1), following 6 weeks of exposure to MTS in heart tissue derived from the same mice [26], further supporting our observations on the lack of inflammation in mice exposed to MTS. PAI-1 is an acute phase response gene, which also plays a role in the inflammation process.

Chronic exposure to cigarette smoke leads to lung inflammation and decreased lung function, and is one of the major risk factors for developing COPD. The lack of inflammation observed in our model could be attributed to the non-chronic nature of the exposure protocol used in the present study. Similar suppression of inflammation in response to smoke has been observed previously. In a comparative study, Meng et al. [67] exposed mice to smoke, lipopolysaccharide (LPS), or a combination of smoke plus LPS, using a nose-only inhalation exposure system. Lung tissues were analyzed using Affymetrix GeneChip microarrays. The authors found up-regulation of a small number of genes involved in inflammation in the smoke-exposed group compared to a more robust response in the LPS group. In addition, the number of neutrophils in BALF was also reduced in smoke-exposed mice. However, pulmonary macrophages and levels of IL-6 were elevated in response to smoke [67]. In another study, Stevenson et al. reported time-dependent changes in the expression of genes involved in stress response and inflammation [68]. These results were further supported by histological alterations and changes in cytokine response. Despite the very prominent inflammatory changes observed in this model, there was no increase in IL-6 level in either BALF or lung homogenates [68]. These results suggest that more acute and chronic exposures are needed to address the role of IL-6 in smoke-induced lung and systemic inflammation.

Several studies have reported that the pro- and anti-inflammatory activities of IL-6 can be modulated by other effectors, including members of the SOCS family. SOCS3 is an IL-6 responsive gene and is a specific inhibitor of the IL-6/Stat3 signalling pathway [69]. Under physiological conditions, the IL-6/JAK/Stat3 pathway induces expression of SOCS3, which then binds tyrosine 759 of membrane protein Gp-130, inhibits activation of JAK-1, and hence blocks IL-6 signalling in a classic feed-back loop [69]. In macrophages, SOCS3 has been shown to regulate the contradictory pro- and anti-inflammatory actions of IL-6. Mutations in the SOCS3 binding site of Gp-130, or complete lack of SOCS3 in macrophages, results in suppression of LPS-induced TNF production by IL-6 [69]. Specific deletion of SOCS3 in macrophages and neutrophils results in resistance to acute inflammation induced by LPS. These results clearly demonstrate the role of SOCS3 in the inflammatory process and suggest that the absence of SOCS3 may contribute to anti-inflammatory activities of IL-6. However, inhibition of SOCS3 is also implicated in tumor progression and malignancy. In human cholangiocarcinoma, sustained IL-6/Stat3 signalling, enhanced Mcl-1 expression and resistance to apoptosis, is attributed to epigenetic silencing of SOCS3 by promoter hypermethylation [70]. SOCS3 is also silenced by hypermethylation in a human lung cancer cell line [71].

In the present study, despite the dramatic increase in mRNA expression of SOCS3 following 12 weeks of MTS exposure, SOCS3 protein levels decreased in total lung extracts, suggesting post-transcriptional regulation of SOCS3 expression. Although we could not confirm the phosphorylation of SOCS3 in our model, SOCS3 phosphorylation has been shown to decrease the half life of the protein [72]. This, in turn, may lead to sustained IL-6 signalling and malignant transformation of cells. In our study, we observed gradual increases in MCL-1 and BCL-XL protein levels, both of which are implicated in cancer progression.

One of the main limitations of the present study is that the microarray experiments were conducted using total RNA from whole lung tissues rather than on individual pulmonary cell types. Consequently, the observed changes in gene expression may reflect changing cell numbers in the lung at the time of sampling. Analysing a single cell type by laser capture microdissection is ideal and may result in a more homogenous response, however, it is also important to understand tissue level effects and how they are related to molecular changes in target cell population [73]. A number of studies have described gene expression changes in various cell types exposed to tobacco smoke, but very few have examined whole lung response. Integration of gene expression profiles with reports examining multiple end points at the molecular, cellular, tissue and physiological levels (i.e., systems biology approaches) are needed to better understand the toxicity in the context of the whole organism [73]. We have also used subchronic levels of MTS exposure that are non-toxic. Our goal was to capture the early changes in gene expression that may potentially be used to predict health outcome of the exposure. More studies incorporating acute, subchronic and chronic doses with multiple time points are needed to validate our findings.

In summary, our results showing IL-6 signalling, coupled with a decrease in SOCS3 protein and no change in total BAL neutrophils, suggest an absence of inflammation in the mouse lungs following 12 weeks of exposure to MTS. We propose that an increase in pro-proliferation and anti-apoptotic molecules, and a decrease in inflammatory and pro-apoptotic proteins such as SOCS3, have succeeded in protecting cells against inflammation during the subchronic exposure used in our study. However, in the event of continued chronic smoking, this balance may be lost, resulting in uncontrolled growth, malignany and cancer development. Alternatively, continued smoking and sustained IL-6 expression may activate other pathways that could ultimately initiate inflammation and contribute to the development of inflammatory lung diseases such as COPD. In general, data presented in this study agree with those described for human smokers. For example, Spira et al. compared cellular material derived from bronchoscopies of chronic smokers, never smokers and former smokers, and showed similar expression changes in xenobiotic metabolism genes [25]. In airway epithelium of smokers with no history of lung disease, in small airway epithelial cells from phenotypically normal smokers and in bronchepithelium of current smokers, genes related to response to xenobiotics and antioxidant genes (e.g., glutathione metabolism and redox balance) were altered [74-76]. Despite the obvious discrepancies between animal and human models, the observed similarities raise hope that animal models provide insight into molecular pathways underlying the effects of tobacco smoke leading to lung disorders.

## Conclusion

We used global gene expression profiling to provide insight into molecular response to a toxicant exposure. Based on our findings, we hypothesize that protection from, or the development of, a disease in response to any toxicant depends on a delicate balance between divergent pathways (inflammatory and anti-inflammatory). Prevalence of one pathway over the other could contribute to the health outcome of an exposure. Characterization of gene expression profiles following tobacco smoke exposure in different models is necessary to identify these divergent pathways and will enhance our understanding of the complexities involved in tobacco smoke-induced molecular pathogenesis.

## Competing interests

There are no financial competing interests to disclose. The authors state that no financial support has been provided by any organization that stands to gain or lose financially by publishing this work. Authors do not hold stocks in any organization that may benefit from this publication. No patents relating to this work is applied for or held.

## Authors' contributions

SH was responsible for the design, planning, and execution of the experiments, and writing the manuscript. MR participated in protein work. MRS was responsible for experimental design, animal exposures and critical review of the manuscript. AW carried out statistical analysis of microarray data. CY was responsible for acquiring funds, experimental design, and critical review of the manuscript. All authors have read and approved the final manuscript.

## Pre-publication history

The pre-publication history for this paper can be accessed here:


